# Food habits and associated risk factors of depressed patients with cardiovascular disease

**DOI:** 10.1371/journal.pone.0263519

**Published:** 2022-02-03

**Authors:** Hind E. Aljuhani, Ghedeir M. Alshammari, Ahmad N. AlHadi, Kholoud B. Alabdulkarem, Omar Sulaiman M. Albader, Mirza B. Baig, Mohammed Abdo Yahya

**Affiliations:** 1 Department of Food Science and Nutrition, College of Food and Agricultural Sciences, King Saud University, Riyadh, Saudi Arabia; 2 Psychiatry Department, College of Medicine, King Saud University Medical City, King Saud University, Riyadh, Saudi Arabia; 3 Department of Social Studies, College of Arts, King Saud University, Riyadh, Saudi Arabia; 4 King Saud Medical City, King Saud University, Riyadh, Saudi Arabia; 5 Prince Sultan Institute for Environmental, Water & Desert Research, King Saud University, Riyadh, Saudi Arabia; Zagazig University, EGYPT

## Abstract

The present study was carried out to investigate food habits and associated risk factors of depressed patients with cardiovascular disease in Riyadh city, Saudi Arabia. Depressed and healthy females (n = 30 each) and males (n = 30 each) aged 18–65 years were involved in this study. Sociodemographic, anthropometric proxies, and nutritional status were evaluated. Cholesterol, triglycerides, high-density lipoprotein cholesterol (HDL-c), and low-density lipoprotein cholesterol (LDL-c) levels of respondents’ blood were determined. The respondents were varied according to demographic factors and anthropometric proxies. The majority of depressed males had higher values than healthy ones. The student t-test analysis showed that the average daily intake of fat especially saturated fat, by depressed respondents was higher than that of the healthy ones as well as the dietary requirement intake (DRI). The analysis of respondents’ blood showed that the number of depressed females had higher abnormal HDL-c than males, who were observed to have an abnormal level of cholesterol and triglycerides. The correlation of daily nutrient intake and depression duration, depression severity, and age showed that the nutrients responsible for the extension and severity of depression were intake of food rich in dietary fat. Factors including demographics daily nutrient intake appeared to be associated with depression.

## Introduction

Major depressive disorder (MDD) is one of the most common psychiatric disorders that can affect a person’s thoughts, behavior, feelings, and sense of well-being [1]. The prevalence of depression and other mental issues has shown that mental illness will prevail among humans [2]. A study was conducted in Saudi Arabia to investigate the relationship between social anxiety disorder (SAD) and depression, concluded that patients with SAD had another current psychiatric disorder and had depression after SAD onset [3]. Naqvi et al. [4] reported that depression increases the risk of coronary heart disease in both men and women and increases coronary heart disease mortality in both, independent of other traditional coronary heart disease risk factors.

The prevalence of major depression is two times higher in women than in men, beginning in early adolescence. This is due to gender differences, including biological factors such as the lifetime of fluctuating hormonal levels from the menstrual cycle and reproduction and psychosocial factors [5]. People with a higher risk of cardiovascular disease are more depressed than the general population because depression and vascular disease are associated with lower coherence (SOC) values, unhealthy lifestyles, and poor sleep quality [6]. In addition to increasing the risk of cardiovascular disease, depression also increases the risk of cardiovascular mortality when cardiovascular disease is already present. Therefore, there is evidence that depression contributes to the onset of cardiovascular diseases and their development and prognosis [7]. A study showed a link between depression symptoms and cardiovascular disease in elderly Americans without primary cardiovascular disease and annual information on their depressive state. It showed that depressive symptoms increase the risk of cardiovascular disease in older adults [8].

Weight gain is increasingly recognized as a medical problem because it occurs concurrently with depressive disorder and is described by a polygenic and heterogeneous factor as a type of pathogenesis [9]. Inappropriate dietary practices have been observed in patients with depressive disorder. They include avoidance of excessive use of certain combinations of items and dishes, which may improve severe health deficiency or abundance of proteins, fats, and sugars [9]. It has been observed that antidepressants increase appetite because the dietary intake will be higher than the permissible [10].

A study reporting cross-sectional relationships of diet, depression, and cardiovascular disease risk indicated evidence of a mediating effect of diet, such that adherence to a Mediterranean diet statistically mediated the relationship between depression scores and cardiovascular disease risk scores [11]. A study by Jacka et al. [12] showed an association between nutrition and depressive disorders increasing interest in recent years. Also, it has been reported that obesity is correlated with an increased risk of mood disorder [13]. A previous study has shown an association between the dietary intake of fatty acid and the increasing risk of depressive disorder [14].

Among women with suspected myocardial ischemia, they observed consistent relationships between depression, dietary habits, and time to cardiovascular disease [15]. Low levels of omega-3 and an imbalance between the ratio of omega-3 to omega-6 were found in depressed patients [16]. Moreover, Frasure-Smith et al. [17], in a case-control study with a post-acute coronary syndrome sample, reported that omega 3 fatty acid and docosahexaenoic acid levels were significantly lower among patients with versus without depression. In addition, the food intake of depressed persons has shown to be less adequate, healthy, and nutritious than that of non-depressed persons.

It has been shown that persons who suffer from depression have a higher 24-h caloric intake than non-depressed ones [18], whereas certain vitamin deficiencies, such as vitamin D, B12, and folate, are more prevalent in depressed persons [19]. Therefore, it is important to note the essential role of fat in the function of neurons, regulation of membrane liquidity, permeability, transport, and release of neurotransmitters [20]. Unhealthy lifestyle habits such as a poor diet may contribute to the development of depression-related physical illnesses [21]. Therefore, this study aimed to investigate food intake and associated risk factors of depressed patients with cardiovascular disease among Saudi females and males.

## Material and methods

### Sample selection and size

A case-control study was conducted in Riyadh city on patients with depressive disorders under care in the medical city in King Saud University, King Salman Social Center, and Edrak Medical Consulting Center. Participants are recruited via advertisements on social media and posters placed at all locations. The study sample consisted of 60 females and males classified by psychologists with depressive disorders (30 females and 30 males aged 18–65), and a healthy group consisted of 60 volunteers (30 females and 30 males aged 18–65) who did not suffer from mental or nutritional disorders or chronic diseases that affect nutrient metabolism. The respondents were randomly selected from a sampling frame using a random numbers table according to a minimum sample size formula. They signed a form according to the Helsinki Declaration. All of the respondents are Saudi.

### Data collection

A structural questionnaire and anthropometric measurements were used for data collection. All participants were provided written informed consent in their native language before enrollment. The questionnaire was validated by committee members from the Department of Nutrition, College of Food and Agriculture, Psychiatry Department, College of Medicine Department of Social Studies, College of Arts, King Saud University. The questionnaire was designed to study sociodemographic factors (education level and social status) and daily food intake (kind of food and amount). Before answering the questionnaire, respondents were given an idea about the study and instructed how to complete the questionnaire truthfully. The 24 hours recall food intake data for each respondent were entered and analyzed using Food Processor Version 11.6 (2019). The software analyzes the food and gives a percentage of all nutrients, including calories, that the respondents have taken. After that, instead of comparing participants to the healthy group’s nutritional status, dietary adequacy was assessed by comparing the participants’ intake with dietary reference intake (DRI) values [22].

### Depression diagnosis

To Screen depression among patients, the PHQ-9 scale was used according to Abdelwahid and Al-Shahrani [23] method. The scale consists of 9 questions answered by the patient to know the severity of depression, as well as the anxiety scale consisting of 7 questions to know the severity of anxiety. The test was done with the help of a psychologist. The excluded people are those who have a known history of damage in the Central Nervous System, and suffering from other mental diseases, people with depression caused by a bipolar affective disorder, have a disease that may harm metabolism parameters, who overuse alcohol, people with high blood sugar levels above 5.18 mmol\L, have fat disorders, are taking treatment to reduce the concentration of fats.

### Anthropometric measurement

Nutritional proxies, including body mass index (BMI kg/m^2^), body fat (BF%), visceral fat (VF), muscle mass (MM) levels, and body water, were determined through bioelectrical impedance analysis (BIA) (MC-780MA, TANITA Corp., Tokyo, Japan). The fluctuation of water content in the body is expected to give an error in body composition; therefore, measurements were taken in the morning when respondents stayed without eating, drinking, showering, or exercising for at least two hours. According to the manufacturer’s instructions, respondents were asked to wipe off the sole of their feet before stepping onto the measuring platform because unclean footpads interfere with the device’s conductivity. BMI (kg/m^2^) was used to assess body weight status. According to the BIA device instruction manual [24], adults were classified according to their BMI as underweight (BMI < 18.5), normal (BMI = 18.5–24.9), overweight (BMI = 25–29.9), or obese (BMI ≥ 30). According to their age, the respondents were classified as having low (BF% < 11), normal (BF% = 11–21.9%), high (BF% = 22–27), or very high (BF% > 27) BF. VF < 10 level = low, 10–14.9 level = normal, 15–20 level = high, and > 20 level = very high.

### Biochemical analysis

Biochemical measurements were made in blood samples of the participants and analyzed in the laboratory of King Khalid University Hospital as well as Aldar Biological Laboratory to measure lipid profile (total cholesterol (TC), triglycerides (TG), high-density lipoprotein (HDL-c), and low-density lipoprotein (LDL-c)).

### Ethics of human subject participation

This study was conducted according to the guidelines laid down in the Declaration of Helsinki. All procedures involving research study participants were approved by the Internal Review Board (IRB) at King Saud University, Riyadh, Saudi Arabia (No: KSU-SE-18-15). All participants provided written informed consent.

### Statistical analysis

Data were analyzed using the Statistical Package for Social Sciences version 22 (SPSS Inc. Chicago, IL, USA). Independent Sample T-test, One-way ANOVA, the Least Significant Difference (LSD) test, and the analysis of variance of repeated measures were used. The correlation between independent variables and dependent ones was tested using Spearman’s correlation coefficients.

## Results and discussion

**Table 1** shows the demographic characteristics and anthropometric proxies of depressed and healthy respondents (females and males). In this study, participants’ selection was based on the fact that the age of respondents should not vary significantly, whether for males or females. Therefore, the mean age of depressed females’ respondents was 45.1 ± 4.8 years, and that of the healthy group was 43.9 ± 5.6 years while the mean age of depressed male respondents was 43.1 ± 4.3 years, and that of the healthy group was 43.3 ± 4.7 years.

**Table 1:** Demographic characteristics and anthropometric proxies of depressed and healthy females and males respondents.

**Variables**	**Females**				**Males**			
	**Depressed n = 30**	**Healthy n = 30**			**Depressed n = 30**	**Healthy n = 30**		
	**%**	**%**			**%**	**%**		
*Education*								
Primary	33.3	6.7			53.3	16.7		
Secondary	50.0	26.7			26.7	40.0		
University	16.7	66.7			20.0	43.3		
*Social status*								
Married	66.7	73.3			73.3	73.3		
Single	33.3	26.7			26.7	26.7		
Variable	Females				Males			
	Depressed	Healthy	t-test	P-value	Depressed	Healthy	t-test	P-value
Age (years)	45.1±4.8	43.9±5.6	0.87	0.387	43.1±4.3	43.3±4.7	0.20	0.842
Height (cm)	159.8±6.1	159.1±5.6	0.44	0.659	173.0±7.1	174.4±5.0	0.88	0.383
Weight (kg)	66.0±17.7	66.3±11.5	0.09	0.929	88.7±19.8	77.4±19.4	2.22	0.031*
BMI	26.7±7.2	26.2±4.3	0.34	0.737	29.9±6.1	25.4±6.1	2.87	0.006*
< 25	22.5±2.7	21.7±1.2	0.95	0.353	22.4±1.7	20.8±2.8	1.23	0.234
> 25	31.5±6.6	29.2±2.7	1.33	0.194	31.8±5.3	30.6±4.4	0.72	0.475
Waist circumference (cm)	88.6±8.9	84.4±7.5	1.95	0.056	102.2±7.0	95.7±10.2	2.84	0.006**
Body fat (kg)	23.2±11.2	22.1±7.7	0.44	0.66	26.2±11.8	18.4±11.2	2.61	0.011*
Body fat (%)	33.5±7.9	32.5±6.2	0.55	0.59	28.1±7.2	21.8±8.6	3.04	0.004**
Visceral fat level	5.2±4.1	4.3±2.6	1.08	0.28	10.8±5.6	6.3±4.3	3.49	0.001**
Muscle mass (kg)	40.6±6.8	42.1±4.6	0.99	0.32	59.3±8.4	56.1±8.1	1.53	0.131
Muscle mass (%)	63.1±7.5	64.0±6.1	0.47	0.64	68.3±6.8	74.2±8.2	3.05	0.003**
Body water (Kg)	30.8±4.9	31.9±3.2	1.08	0.28	44.4±5.9	42.5±5.9	1.29	0.203
Body water (%)	48.0±6.4	48.6±4.4	0.47	0.64	51.3±6.1	56.4±7.3	2.97	0.004**
ECW (Kg)	13.8±2.7	14.0±1.7	0.30	0.76	19.0±2.5	17.7±2.5	1.95	0.057
ECW/TBW (%)	44.6±2.5	43.7±1.6	1.68	0.10	42.7±1.6	41.7±1.2	2.89	0.005**
ICW (kg)	17.0±2.4	18.0±1.6	1.78	0.08	25.5±3.6	24.8±3.5	0.77	0.442
ICW/TBW (%)	55.4±2.5	56.3±1.6	1.67	0.10	57.3±1.6	59.2±5.1	1.97	0.053

Values are means ± SD.

** P ≤ 0.01

* P ≤ 0.05. ECW, extracellular water; ICW, intracellular water.

The study showed that the majority of depressed females and males had a low level of education. The effect of educational level as a major depressive disorder (MDD) is extensively studied. The majority of the studies reported that a higher level of education is inversely proportional to the prevalence of MDD among depressed individuals [25–28]. Alonso et al. [26] have shown that higher education levels are associated with a low rate of MDD among the tested patients. Also, a previous study conducted by Lu et al. [27] in china showed a strong correlation between the fewest years of education and the prevalence of MDD in tested patients. However, no association between educational levels and the prevalence of depression has been reported in the study of Lindeman et al. [29] in Finland patients. Different studies showed that gender, age, and race could potentially impact the association between the educational level and depression disorders among patients [30, 31].

The percentage of depressed females and males was high among married compared to single respondents. It has been reported that self-report measures of depression symptoms and marital dissatisfaction are significantly correlated for both men and women in community samples [32]. Individuals dissatisfied with their relationships are more likely to be clinically depressed than those in satisfactory relationships. Marital dissatisfaction may not only increase vulnerability to depression but may also negatively affect the course and resolution of depression [32].

As shown in **Table 1**, the anthropometric proxies of depressed and healthy females and males respondents were varied between the respondents of both sexes. The results showed that the height and weight of depressed and healthy females were not varied. However, depressed males had weights significantly higher than the healthy ones. The BMI, waist circumference, body fat, visceral fat level, muscle mass, body water, extracellular water (ECW), and intracellular water (ICW) of depressed and healthy females were not significantly different, but they were observed to be significantly higher in depressed respondents’ males than the healthy ones.

The present study showed that most anthropometric proxies, especially for depressed males, were higher than healthy ones. This finding agrees with a report showing that an increase in waist circumference, BMI, and obesity caused an increase in depression, especially for males [13]. This could be explained by the dietary habit where depressed males intake more fats, which leads to visceral and abdominal obesity and subsequently activation of systemic inflammation that leads to depression [13]. However, whether obesity causes depression or depression triggers obesity [33]. Therefore, based on the present data, it is still difficult to determine whether obesity leads to depression for respondents or not.

Although age was shown to be a significant contributing factor in the development of depression [33], the present data suggest that age didn’t contribute significantly to depression in healthy males. However, Takeuchi et al. [34] have shown that age and waist circumference are the most contributing factors leading to depression in patients with metabolic syndrome. Moreover, Zhao et al. [13] reported that abdominal obesity and visceral fats activate various inflammatory pathways and mediators, resulting in depression. Therefore, these observations in previous studies could explain the increase in BMI, waist circumferences, and body weight in depressed male patients. However, normal BMI, waist circumferences, and body weight was observed in depressed females despite the high intake of MUFA and SFA. A previous study showed that high dietary intake of cholesterol and higher serum cholesterol levels, especially low-density lipoprotein (LDL-c), are positively correlated with BMI [35].

In this study, the average dietary intake and the energy values for all participants were analyzed using Food Processor version 11.6. The daily food intake of depressed and healthy females and male respondents are shown in **Table 2**. For both females and males, the intake of calories was not significantly different between depressed and healthy respondents. The intake of protein, dietary fiber, and polyunsaturated fatty acids (PUFA) by healthy respondents of both groups was significantly (P ≤ 0.01) higher than that of depressed ones. The intake of carbohydrates by a healthy female was significantly (P ≤ 0.01) higher than that of depressed ones. The depressed respondents of both groups significantly (P ≤ 0.01) had a higher intake of total fat, saturated fatty acids (SFA), monounsaturated fatty acids (MUSFA), and their energy than the healthy group. Other food nutrients were varied between the groups, but the difference was not significant.

**Table 2:** Daily nutrients intake of depressed and healthy females and males respondents.

Variables	Females				Males			
	Depressed n = 30	Healthy n = 30	t-test	P-value	Depressed n = 30	Healthy n = 30	t-test	P-value
Calories	1750.8±222.1	1703.6±201.8	0.85	0.396	1888.1±235.3	1893.3±180.2	0.10	0.923
Total protein (g)	66.7±7.8	75.5±9.3	4.01	<0.001**	72.4±8.8	86.4±10.6	5.56	<0.001**
Total protein (%)	16.2±1.5	19.1±2.7	5.20	<0.001**	16.3±2.1	16.5±2.5	0.44	0.664
Carbohydrate (g)	234.0±23.2	245.9±31.7	1.65	0.103	239.5±26.4	286.8±23.5	7.33	<0.001**
Carbohydrate (%)	52.7±2.8	54.2±2.8	2.13	0.038*	49.8±1.9	52.1±3.2	3.44	0.001**
Dietary Fiber(g)	17.0±3.0	20.6±3.3	4.47	<0.000**	17.2±2.3	21.8±3.2	6.32	<0.001**
Total Fat (g)	65.4±11.6	52.4±9.5	4.73	<0.000**	78.6±11.2	72.1±9.0	2.47	0.017*
Energy (total fat)%	31.9±3.4	28.2±3.2	4.32	<0.000**	36.1±5.1	31.6±3.2	4.11	<0.001**
Cholesterol (mg)	282.9±82.8	271.9±62.9	0.58	0.566	306.3±57.2	274.8±48.1	2.31	0.025*
SFA (g)	25.4±5.6	20.9±5.5	3.15	0.003**	34.5±6.5	30.3±4.7	2.85	0.006**
Energy (SFA)%	14.1±1.4	11.1±1.8	7.24	<0.001**	16.1±1.6	13.4±2.4	5.06	<0.001**
MUFA (g)	24.7±5.4	19.3±3.6	4.56	<0.001**	30.0±5.4	27.8±4.8	1.70	0.094
Energy(MUFA)%	13.6±2.3	11.8±2.2	3.12	0.003**	14.1±1.9	13.6±1.9	1.16	0.250
PUFA (g)	6.8±2.0	8.8±2.1	3.80	<0.001**	7.1±2.0	10.7±2.9	5.48	<0.001**
Energy (PUFAT)%	3.7±0.9	4.4±1.3	2.14	0.037*	3.2±0.6	4.5±1.0	6.17	<0.001**
Trans fatty acid (g)	1.5±2.4	1.2±2.2	0.57	0.569	2.0±2.9	1.6±2.3	0.62	0.537
n-3g	0.7±0.2	0.7±0.2	1.22	0.228	0.8±0.2	0.7±0.2	0.27	0.792
n-6g	5.0±1.6	4.9±1.7	0.29	0.776	6.0±2.3	5.9±1.6	0.33	0.741
n6\n3	7.6±0.8	7.0±1.2	2.30	0.025*	8.0±2.0	8.1±1.0	0.28	0.780
Vitamin D (mcg)	1.3±1.9	2.0±2.3	1.17	0.247	0.5±0.7	0.6±0.8	0.38	0.705
Calcium (mg)	491.9±49.5	535.7±53.7	0.55	0.584	438.4±68.0	394.8±36.1	0.67	0.506
Vitamin A (RE)	648.1±41.8	588.87±25.9	0.69	0.492	537.54±83.9	607.92±91. 9	0.80	0.429
Vitamin -B1(mg)	1.10±0.5	1.22±0.4	1.02	0.311	1.04±0.6	1.09±0.5	0.36	0.722
Vitamin-B2 (mg)	3.33±4.3	3.42±3.7	0.09	0.927	1.29±0.8	1.14±0.6	0.81	0.421
Vitamin-B3 (mg)	12.84±7.2	19.86±8.3	3.51	0.001	12.74±8.0	16.44±9.3	1.65	0.105
Vitamin-B12 (mg)	2.01±1.3	2.54±1.2	1.70	0.095	2.19±1.9	1.61±1.1	1.48	0.145
Vitamin-B6 (mg)	2.49±0. 7	2.67±0.6	1.06	0.295	2.40±0.1	2.72±0.6	1.54	0.130

Values are means ± SD.

** P ≤ 0.01

* P ≤ 0.05.

Further, the average intake of a nutrient of depressed respondents was compared to the average values recommended by the DRI using the t-test. **Table 3** shows the relation of average daily nutrient intake (24-h recall) with the DRI for the respondents. The average calorie intake for both females and males was significantly (P ≤ 0.01) lower than that recommended by the DRI. The amount of protein, carbohydrates, saturated fatty acids, and vitamins consumed by both groups was significantly (P ≤ 0.01, P ≤ 0.05) higher than that recommended by the DRI. Depressed males take significantly (P ≤ 0.01) higher amounts of total fat than that recommended by the DRI. For both groups, the amount of dietary fiber consumed was significantly (P ≤ 0.01) lower than that recommended by the DRI.

**Table 3:** Nutrients intake of depressed males and females compared to daily-recommended intake (DRI).

Nutrients	Females			Males		
	Mean ± SD	DRI	*p*-Value	Mean ± SD	DRI	*p*-Value
Calories	1750.8±222.1	2000	<0.001**	1888.1±235.3	2000	0.014*
Total protein (g)	66.7±7.8	32	<0.001**	72.4±8.8	32	<0.001**
Total carbohydrate (g)	234.0±23.2	130	<0.001**	239.5±26.4	130	<0.001**
Dietary fiber (g)	17.0±3.0	28	<0.001**	17.2±2.3	28	<0.001**
Total fat (g)	65.4±11.6	65	0.837	78.6±11.2	65	<0.001**
SFA (g)	25.4±5.6	20	<0.001**	34.5±6.5	20	<0.001**
Cholesterol (mg)	282.9±82.8	300	0.267	306.3±57.2	300	0.549
Vitamin-A(mg)RE	648.10±411.8	550	0.202	537.54±283.9	550	0.812
Vitamin-B1(mg)	1.10±0.5	0.800	0.005**	1.04±0.6	0.800	0.029*
Vitamin-B2(mg)	3.33±4.3	0.800	0.003**	1.29±0.8	0.800	0.002**
Vitamin-B3(mg)	12.84±7.2	11	0.170	12.74±8.0	11	0.245
Vitamin-B12(mg)	2.01±1.3	0.90	<0.001**	2.19±1.9	0.90	0.001**
Vitamin-B6(mg)	2.49±0. 7	1.60	<0.001**	2.40±0.1	1.6	<0.001**

Values are means ± SD.

** P ≤ 0.01

* P ≤ 0.05.

As demonstrated in this study, a high intake of total fat, saturated fatty acids, and unsaturated fatty acids by depressed females and males compared to healthy respondents and a high intake of saturated fats compared to DRI could be considered severe harmful habits. It has been reported that the increased dietary intake of a high-fat diet is a severe risk factor for the development of depression among males and females [14].

The dietary regimen of both males and females patients with depression is characterized by a low intake of carbohydrates, protein, and fibers. However, it involves a high intake of dietary fats especially saturated fatty acids, as reported by many researchers [18, 36]. Further, the present study supported the findings of Grossniklaus et al. [37], Appelhans et al. [36], and Ljungberg et al. [38], who reported that the severity of depression symptoms is increased with high dietary fat intake. Moreover, an abnormal energy intake with reduced calorie intake from protein but higher in fats was observed in the diet of a patient with depression [39]. Also, a stable level of cholesterol compared to DRI was observed by Wendołowicz et al. [39], who have shown that the average daily intake of cholesterol did not exceed the DRI (>300mg/day) in both males and females patients with depressive disorder.

It has been observed that a diet rich in n-6 PUFA and poor in n-3 PUFA is an independent risk factor for obesity, depression, cerebrovascular, cardiovascular (CVD), and inflammatory disorders [40, 41]. However, during the last 4 decades, a significant shift toward a diet rich in n-6 and poor in n-3 has been developed among our societies and was coincided with the sudden increase in psychological disorders, depression, CVDs, and other chronic disorders [40]. The major forms of omega 3 PUFA in our diet are those of marine sources, including eicosapentaenoic acid (EPA) and docosahexaenoic acid (DHA) as well as alpha-linoleic acid from plants, whereas linoleic acid (LA) is the major form of n-6 PUFA in plants such as sunflower, safflower, and corn oils [40]. Despite the unaltered n-3 and n-6 PUFA levels in the serum of depressed males and females in this study, the significant increase in the ratio of n-6/n-3 PUFA in females may contribute significantly to the development of depression among those females.

The lipid analysis in respondents’ blood showed that 36% of depressed females had an abnormal cholesterol level versus 10% of healthy ones (**Table 4**). However, 53.3% of depressed males versus 6.7% of healthy ones had an abnormal cholesterol level. The abnormal level of triglycerides in depressed males was higher than that of females. High-density lipoprotein cholesterol (HDL-c) abnormal level in females, whether depressed or healthy, was higher than that of males. However, low-density lipoprotein cholesterol (LDL-c) abnormal levels in males, whether depressed or healthy, were higher than in females.

**Table 4:** Cholesterol, triglycerides, high-density lipoproteins cholesterol (HDL-c), and low-density lipoproteins cholesterol (LDL-c) levels of respondents.

Variables		Females				Males			
		Depressed n = 30		Healthy n = 30		Depressed n = 30		Healthy n = 30	
		N	%	N	%	N	%	N	%
Cholesterol (mmol/L)	Normal (<5.2)	19	63.3	27	90.0	14	46.7	28	93.3
	Abnormal (>5.2)	11	36.7	3	10.0	16	53.3	2	6.7
Triglyceride (mmol/L)	Normal (<1.7)	24	80.0	29	96.7	9	30.0	25	83.3
	Abnormal (>1.7)	6	20.0	1	3.3	21	70.0	5	16.7
HDL-c (mmol/L)	Normal (<1.5)	13	43.3	13	43.3	29	96.7	26	86.7
	Abnormal (>1.5)	17	56.7	17	56.7	1	3.3	4	13.3
LDL-c (mmol/L)	Normal (<2.6)	8	26.7	13	43.3	5	16.7	12	40.0
	Abnormal (>2.6)	22	73.3	17	56.7	25	83.3	18	60.0

The nature of the association between depression and CVD remains to be elucidated. However, it has been suggested that both depression and CVD stem from the exact pathological cause, altered cholesterol metabolism, including high levels of LDL-c and the parallel decrease in HDL-c [42]. The increased circulatory levels of LDL-c and oxidized LDL-c are considered the major contributors for developing ischemic heart disease and atherosclerosis by triggering immunological cascades at the endothelial walls of the blood vessels that end up with foaming cells formation [42]. However, the higher levels of circulatory HDL-c are considered protective against cardiovascular disease. They have anti-inflammatory properties and can inhibit the oxidation of LDL-c and remove cholesterol from the foaming cells [43].

HDL-c was observed to be significantly decreased, but the level of LDL-c increased in patients with CVD [42]. Studies showed that patients with MDD exhibit a significant decrease in the circulatory levels of HDL-c with a significant increase in circulatory levels of LDL-c and LDL-c/HDL-c ratio, which mediated depression by increasing circulatory levels of inflammatory cytokines including interleukin-6 (IL-6) and tumor necrosis factor-alpha (TNF-α) [44–46]. Several studies have demonstrated that a low level of total serum cholesterol in depressed patients as compared to healthy was associated with reduced synaptic plasticity and altered membrane receptors, especially serotonin (5-HT) receptors [47, 48]. In this study, depressed males and females’ patients had high circulatory levels of cholesterol, triglycerides, and LDL-c. A high serum level of cholesterol and LDL-c is expected to be true risk factors and major mechanisms that underlie the pathogenesis of both depressions with cardiovascular disorders (CVDs) in those patients.

To investigate the determinant factors of depressed Saudi females and males, Spearman correlation coefficients between the respondents’ illness duration, depression severity, age, and daily nutrient intake were calculated (**Table 5**). The results showed that most independent variables were positively or negatively correlated with the dependent variables, but the strength of correlation varied. The results showed that total fat, saturated fat, and HDL-c were significantly and positively correlated with illness duration and severity of depressed females and males respondents. Among depressed females, the level of triglycerides (P ≤ 0.01) was significantly and positively correlated with illness duration. However, among the male group, the level of triglycerides was positively correlated with illness duration, but the strength of correlation was not significant. Intake of vitamin D and calcium were significantly (P ≤ 0.05) and negatively correlated with depressed females’ age. Intake of PUFA and PUFA energy were significantly (P ≤ 0.05) and negatively correlated with depressed males’ age, but the intake of fiber was significantly (P ≤ 0.01) and positively correlated. Another nutrient intake was either positively or negatively correlated with illness duration, depression severity, and age, but the correlations were not significant. For both sexes, fats and fat derivatives were risk factors for illness duration and severity.

**Table 5:** Spearman correlation between nutrients intake, serum lipid parameters and duration of illness, the severity of depression (PHQ-9), and age of depressed respondents.

Dependent variable / Independent variable	Female						Male					
	Illness duration		Depression severity		Age		Illness duration		Depression severity		Age	
	r	p-value	r	p-value	r	p-value	r	p-value	r	p-value	r	p-value
Total fat (g)	0.04	0.83*	0.04	0.84*	0.29	0.12	0.28	0.13*	0.31	0.09*	-0.08	0.69
Fat energy (%)	-0.05	0.78	-0.05	0.79	0.11	0.56	-0.27	0.14	-0.14	0.46	-0.09	0.65
SFA (g)	0.17	0.36*	0.15	0.42**	0.14	0.45	0.18	0.35*	0.04	0.83**	0.03	0.88
SFA energy (%)	-0.05	0.80	0.14	0.48	0.25	0.18	-0.17	0.37	-0.13	0.50	0.22	0.25
Total MUFA (g)	0.22	0.24	-0.10	0.60	0.04	0.84	0.10	0.61	0.20	0.28	-0.06	0.76
MUFA energy (%)	-0.07	0.73	0.30	0.11	0.27	0.14	-0.06	0.75	-0.08	0.67	-0.27	0.15
PUFA (g) Total	0.10	0.61	0.15	0.44	0.28	0.13	0.04	0.82	0.07	0.72	-0.38	0.038*
PUFA energy %	0.03	0.88	0.00	1.00	0.26	0.17	-0.27	0.15	-0.22	0.24	-0.41	0.023*
Cholesterol Mg	-0.08	0.67	0.16	0.40	0.33	0.08	-0.25	0.18	-0.29	0.12	-0.19	0.31
n6/n3	0.06	0.76	0.18	0.35	-0.33	0.07	0.09	0.65	-0.13	0.49	0.31	0.10
Dietary Fiber(g)	-0.17	0.37	-0.15	0.44	-0.32	0.09	-0.24	0.21	-0.19	0.32	0.49	0.006**
Vitamin D mcg	-0.10	0.58	0.08	0.67	-0.41	0.024*	0.16	0.41	-0.17	0.37	0.18	0.35
Calcium-mg	-0.16	0.39	0.15	0.43	-0.42	0.019*	-0.06	0.76	-0.09	0.63	-0.03	0.89
Cholesterol (mmol/L)	0.14	0.44	-0.28	0.13	0.06	0.74	0.06	0.74	-0.03	0.88	0.22	0.24
Triglyceride (mmol/L)	0.47	0.009**	0.42-	0.022	0.25	0.18	0.15	0.43	-0.17	0.37	0.21	0.26
HDL (mmol/L)	0.40	0.027*	0.10	0.61*	-0.12	0.52	0.27	0.15*	0.06	0.76*	-0.11	0.57
LDL (mmol/L)	-0.27	0.15	-0.34	0.07	0.03	0.86	0.02	0.93	0.11	0.55	-0.21	0.26

** P ≤ 0.01

* P ≤ 0.05.

## Conclusion

In this study, the results indicated that the majority of depressed females and males had a low level of education. The percentage of depressed females and males was high among married compared to single respondents. Most anthropometric proxies, especially for depressed males, were higher than healthy ones. A high intake of total fat, saturated fatty acids, and unsaturated fatty acids by depressed females and males compared to healthy respondents and DRI. Depressed males and females’ patients had high circulatory levels of cholesterol, triglycerides, and LDL-c. For both sexes, fats and fat derivatives were risk factors for illness duration and severity.

## Limitations

The results are not representative of the entire population of Saudi Arabia. Besides, it was a case-control study done at specific locations in Riyadh city. Difficulty in dealing with some patients to convince them to contribute to this study. Participants’ selection was based on the fact that the age of respondents should not vary significantly, whether for males or females. The study was conducted at the height of the COVID-19 outbreak, which limited our movements.
